# Increased presepsin levels are associated with the severity of fungal bloodstream infections

**DOI:** 10.1371/journal.pone.0206089

**Published:** 2018-10-31

**Authors:** Yuuki Bamba, Hiroshi Moro, Nobumasa Aoki, Takeshi Koizumi, Yasuyoshi Ohshima, Satoshi Watanabe, Takuro Sakagami, Toshiyuki Koya, Toshinori Takada, Toshiaki Kikuchi

**Affiliations:** Department of Respiratory Medicine and Infectious Diseases, Niigata University Graduate School of Medical and Dental Sciences, Niigata, Japan; Institute of Microbiology, SWITZERLAND

## Abstract

**Background:**

Presepsin is a widely recognized biomarker for sepsis. However, little is known about the usefulness of presepsin in invasive fungal infection. The aim of this study was to determine the plasma levels of presepsin in fungal bloodstream infections and to investigate whether it reflects the disease severity, similar to its utility in bacterial infections.

**Methods:**

We prospectively measured presepsin in plasma samples from participants with fungemia from April 2016 to December 2017. The associations of C-reactive protein, procalcitonin, and presepsin concentrations with the severity of fungemia were statistically analyzed. *In vitro* assay was performed by incubating *Escherichia coli*, *Candida albicans*, and lipopolysaccharide to whole blood cells collected from healthy subjects; after 3 h, the presepsin concentration was measured in the supernatant and was compared among the bacteria, fungi, and LPS groups.

**Results:**

Presepsin was increased in 11 patients with fungal bloodstream infections. Serial measurement of presepsin levels demonstrated a prompt decrease in 7 patients in whom treatment was effective, but no decrease or further increase in the patients with poor improvement. Additionally, presepsin concentrations were significantly correlated with the Sequential Organ Failure Assessment score (r = 0.89, p < 0.001). *In vitro* assay with co-incubation of *C*. *albicans* and human whole blood cells indicated that the viable cells of *C*. *albicans* caused an increase in presepsin, as seen with *E*. *coli*.

**Conclusions:**

Plasma presepsin levels increased in patients with fungal bloodstream infection, with positive association with the disease severity. Presepsin could be a useful biomarker of sepsis secondary to fungal infections.

## Introduction

The number of immunocompromized hosts has been increasing due to the advances in transplantation medicine and anticancer therapy and the expansion of the use of biological preparations. Within this context, invasive fungal infections (IFIs) are becoming more important diseases. Because IFIs are rare but fatal diseases, clinicians are struggling with the diagnosis and treatment of such conditions. Among the IFIs, candida infection is the most common, especially in the healthcare setting [1, 2]. Invasive candidiasis has high rates of mortality and morbidity [2]. Therefore, early diagnosis of candida infection and prompt initiation of appropriate treatment are important.

Several studies have been conducted to develop biomarkers for the diagnosis and prediction of the severity of candidemia and invasive candidiasis. Candida mannan and anti-mannan antibody are often used for the diagnosis of candidiasis. In a meta-analysis, sensitivity and specificity of mannan and anti-mannan IgG for the diagnosis of the disease were reported to be 58% and 93%, and 59% and 83%, respectively [3]. (1,3)-β-D-glucan, which is a cell wall constituent of several fungi, is commonly used for the diagnosis of fungal infection and has been shown by a meta-analysis to have sensitivity of 75% and specificity of 87% for the diagnosis of IFIs [4]. Leon et al. reported that reduction of (1,3)-β-D-glucan levels during antifungal therapy was associated with treatment success [5]. However, information on the utility of (1,3)-β-D-glucan and the other laboratory findings as prognostic markers of IFIs is limited.

Presepsin (P-SEP) is a soluble CD14 subtype that was reported to be a novel biomarker in sepsis [6]. An increasing number of studies have shown that P-SEP is not only useful for the diagnosis of sepsis but was also predictive of the severity and mortality of the disease [6–9]. In the meta-analysis evaluating the efficacy of P-SEP, the pooled sensitivity and specificity for diagnosing sepsis were 86% and 78%, respectively [10]. The median cutoff value in these studies was 560 pg/mL (IQR 317–729). Procalcitonin (PCT) is also expected as a diagnostic marker for sepsis, and in the meta-analysis, it is reported that the median cutoff value is 1.1 ng/mL (IQR 0.5–2.0) [11]. However, these studies primarily focused on bacterial infection, and little is known about the efficacy of P-SEP in fungal infections. In addition, information on the production mechanism of P-SEP is still limited [12]. The study demonstrated that P-SEP production was triggered by phagocytosis.

We hypothesized that P-SEP levels increase in fungal bloodstream infections by means of phagocytosis and that this increase reflects the severity of the disease, similar to the observation in bacterial infections. First, we examined the plasma P-SEP levels in patients with fungal bloodstream infection. Next, we performed an *in vitro* study to compare the increase in P-SEP levels between fungi and bacteria.

## Materials and methods

### Study design

We performed a prospective study on consecutive patients who were hospitalized for fungemia at the Niigata University Medical & Dental Hospital from April 2016 to December 2017. Day 1 was defined as the time when the positive blood culture samples were drawn and collected. The residual or preserved plasma samples were stored at −80°C. The clinical data collected from the electronic medical records included age, sex, medical history, and laboratory test results. We used the earliest examination results measured after blood culture sampling. Sequential Organ Failure Assessment (SOFA) scores were based on these measured values. Baseline SOFA scores were calculated using the latest values measured during outpatient examination. Plasma P-SEP was measured using a rapid chemiluminescent enzyme immunoassay (PATHFAST immunoanalyzer; LSI Medience Corporation, Tokyo, Japan). The PCT levels in the specimens were analyzed by an electrochemiluminescence immunoassay (SRL, Inc., Tokyo, Japan). Cutoff levels for PCT and presepsin were set at 500 pg/mL and 0.5 ng/mL, respectively, according to the manufactures' protocols [13, 14]. This study complied with the principles of the Declaration of Helsinki and the current ethical guidelines and was approved by the ethics committee of Niigata University Medical and Dental Hospital (#2015–2432).

### *In vitro* assay

Heparinized human whole blood was collected from 6 healthy male volunteers of our hospital and university staff (mean age was 30.0 years) using Venoject II blood collection tubes (Terumo, Tokyo, Japan). We used *Candida albicans* strains isolated from patient #8; the control assay contained *Escherichia coli* strains (American Type Culture Collection number 25922), which were stored at −80°C using a Microbank system (Pro-lab Diagnostics, Austin, Texas, USA). Colony forming units (CFUs) were calculated using a spread plate technique at ten-fold dilution and incubation for 24 h at 37°C. *E*. *coli* was cultured in Mueller–Hinton broth (Becton Dickinson, East Rutherford, NJ) for 24 h, and the *C*. *albicans* in Sabouraud–Dextrose broth (Becton Dickinson, East Rutherford, NJ) was diluted 2 × 10^8^ CFU/mL with sterilized PBS. Lipopolysaccharide (LPS, Wako, Osaka, Japan) was diluted with each liquid medium to a concentration of 200 μg/mL. Each solution of 50 μL of *E*. *coli*, *C*. *albicans* (1 × 10^7^ CFU), and 10 μg LPS in Mueller–Hinton or Sabouraud–Dextrose broth was added separately to 1 mL of whole blood and incubated at 37°C for 180 min. After centrifugation at 3000 rpm for 10 min, the P-SEP in the supernatant was measured and compared among the solutions.

We conducted similar experiments with other *Candida* species (*C*. *tropicalis*, *C*. *guilliermondii*, *C*. *parapsilosis*, and *C*. *glabrata*) isolated from patients #2, #3, #6, and #10. Five species of *Candida*, including *C*. *albicans*, were cultured and diluted according to the abovementioned method. To 1 mL of whole blood from 4 other healthy university staff volunteers (mean age was 31 years), 50 μL of each solution (1 × 10^7^ CFU) was separately added and incubated at 37°C for 180 min. After centrifugation, the P-SEP in the supernatant was measured and compared.

### Statistical analysis

We used the Shapiro–Wilk test to determine whether the values of the parameters were distributed normally. In the *in vitro* assay, paired t-test was used for comparison between 2 groups. Correlations between plasma P-SEP and the clinical data were analyzed using the Spearman’s rank correlation test. To examine the relationships between each biomarker and patient death, we produced ROC curves after generating a univariate logistic regression model. A value of p < 0.05 was considered statistically significant. All data were analyzed using the JMP 13 (SAS Institute Inc., NC, USA).

## Results

### Patient characteristics

The background characteristics of the 11 participants with fungemia are shown in Table 1. Four of the 11 patients died despite intensive treatment, including antifungal agents. Patients #4 and #11 had complications of bacterial infection. Patient #8 received antibiotic treatment for 79 days and had good progress until the onset of fungemia.

**Table 1:** Background characteristics of 11 patients with fungal bloodstream infection.

	Age (years)	Sex	Isolates	Underlying diseases	Antifungal drugs	Clinical outcomes
1	88	F	*C*. *tropicalis*	extensive burn	MCFG/CPFG/FLCZ/L-AMB	survived
2	74	M	*C*. *tropicalis*	post treatment of invasive pneumococcal infection	MCFG	died (day 50)
3	37	F	*C*. *guilliermondii*	ulcerative colitis	F-FLCZ	survived
4	80	M	*C*. *albicans*	diabetes, peripheral arterial disease, infectious leg ulcer*	MCFG	survived
5	74	F	*C*. *parapsilosis*	ulcerative colitis, diabetes	F-FLCZ	survived
6	35	F	*C*. *parapsilosis*	intestinal pseudoobstruction	F-FLCZ	survived
7	41	F	*C*. *tropicalis*	acute myelogenous leukemia (post cord blood cell transplantation and remission), drug-induced lung injury, diabetes, chronic renal failure	MCFG	died (day 1)
8	80	M	*C*. *albicans*	pyogenic spondylitis, iliopsoas abscess	MCFG/FLCZ/L-AMB	died (day 81)
9	34	M	*Trichosporon spp*.	acute lymphoid leukemia, chronic renal failure, chronic heart failure, neutrophil aplasia	CPFG/L-AMB	died (day 4)
10	51	F	*C*. *albicans*, *C*. *glabrata*	systemic lupus erythematosus, short bowel syndrome	MCFG	survived
11	75	F	*C*. *albicans*, *E*. faecium*	post treatment of ventilator-associated pneumonia, chronic heart failure, chronic renal failure (on hemodialysis)	MCFG	survived

*Concomitant active bacterial infection was present in 2 of 11 patients.

MCFG; Micafungin, CPFG; Caspofungin, FLCZ; Fluconazole, F-FLCZ; Fosfluconazole, L-AMB; Liposomal amphotericin B.

The characteristics and laboratory data are summarized in Table 2. The median P-SEP concentration was >500 pg/mL, whereas the PCT levels were only slightly increased. Only five patients had PCT levels above 0.5 ng/mL, two of which were concomitant with active bacterial infection (S1 Table). Focusing on sepsis patients, P-SEP increased (>500 pg/mL) in all 6 patients; however, the increase in PCT (>0.5 ng/mL) was observed in 4 of the 6 patients. In patient #7, who received treatment with intravenous methylprednisolone at 1 g per day for 3 consecutive days, P-SEP was markedly increased at 9,367 pg/mL, whereas CRP was only slightly increased at 3.04 mg/dL. Serum (1,3)-β-D-glucan measured as late as day 7 in all patients, except patients #6 and #8, and its cutoff level was set at >11 pg/mL, as suggested by the manufacturer. In all patients, serum (1,3)-β-D-glucan was >11 pg/mL.

**Table 2:** Characteristics and laboratory data of 11 patients with fungemia.

Characteristics	Value
Age (years)	74 (37–80)
Men (%)	5 (45)
SOFA score*	3 (1–10)
Sepsis^†^ (%)	6 (55)
Patients who died (%)	4 (36)
Laboratory Data	Value
WBC (/μL)	10764 ± 8337
Neutrophil (/μL)	8881 ± 7129
Lymphcyte (/μL)	1285 ± 1630
Eosinophil (/μL)	60 ± 70
Basophil (/μL)	24 ± 28
Monocyte (/μL)	513 ± 621
Albumin (g/dL)	2.8 ± 0.6
Hemoglobin (g/dL)	9.3 ± 1.4
Platelet (×10^4^/μL)	17.7 ± 10.5
Creatinine (mg/dL)	1.7 ± 1.3
Total bilirubin (mg/dL)	0.7 (0.3–1.4)
(1,3)-β-D-glucan^‡^	33.5 (17.1–183.7)
CRP (mg/dL)	8.6 (0.2–16.6)
PCT (ng/mL)	0.5 (0.1–11.5)
P-SEP (pg/mL)	975 (748–3591)

Data are presented as mean ± SD, or median (interquartile range).

SOFA score, sequential organ failure assessment score; WBC, white blood cell count; CRP, C-reactive protein; PCT, procalcitonin; P-SEP, presepsin.

*SOFA scores, based on six different scores, one each for the respiratory, cardiovascular, hepatic, coagulation, renal and neurological systems, were defined on laboratory data on day 1 in 9 patients and on day 2 in 2 patients (patient #8 and #11).

^†^Sepsis was defined with an increase of 2 or more points from the baseline in SOFA score.

^‡^Serum (1,3)-β-D-glucan were measured as late as day 7 in all patients, except #6 and #8. The earliest data were shown.

### Relationship of C-reactive protein, procalcitonin, and presepsin concentrations with the severity of fungemia

**Fig 1:**
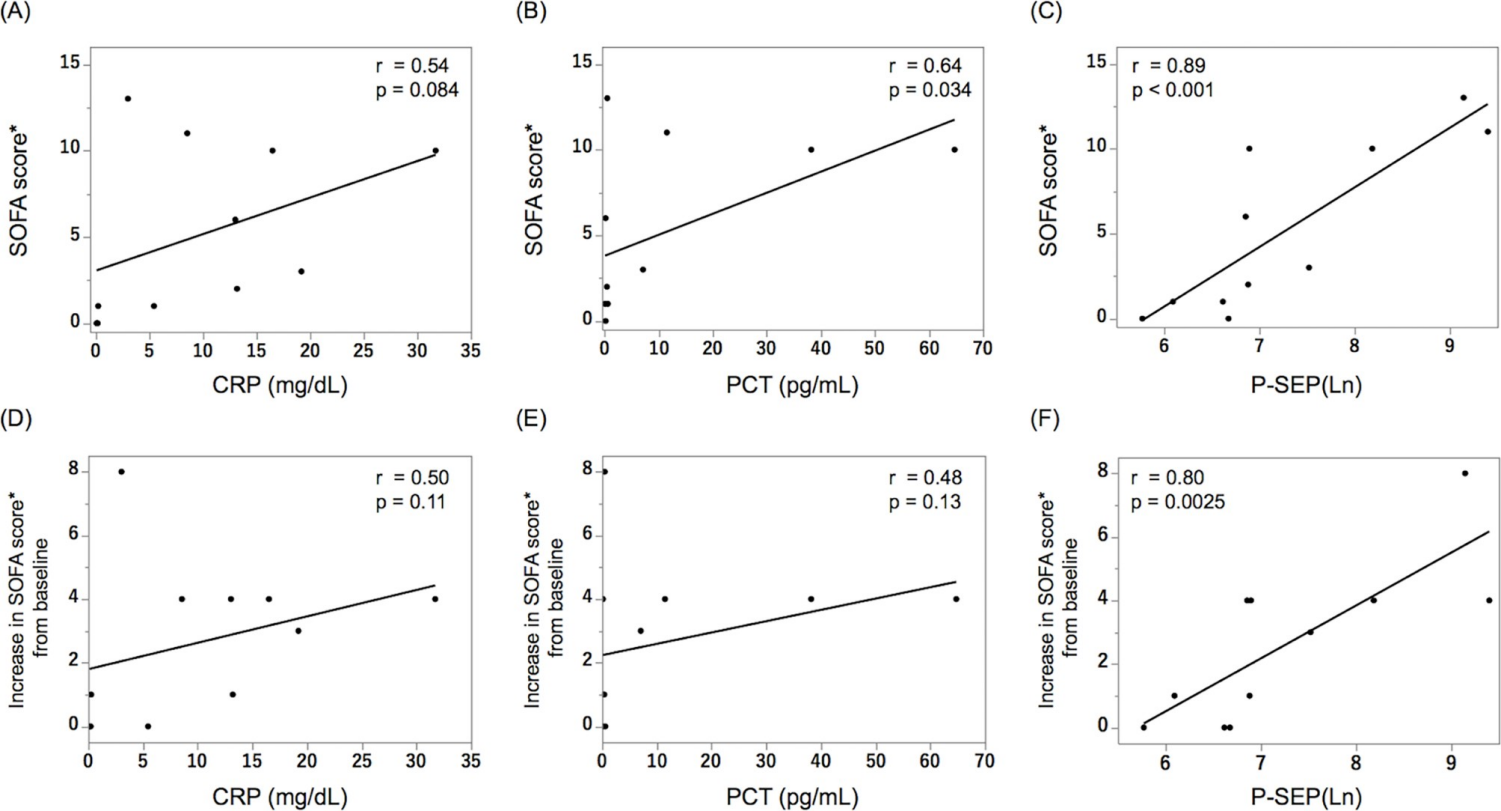
Relationship of the CRP, PCT, and P-SEP concentrations with the SOFA scores. There is a significant association between the PCT levels and the SOFA scores before treatment, but not between CRP and SOFA scores (A, B). The SOFA scores have the strongest positive correlation with the P-SEP concentrations (r = 0.89, p < 0.001, C). The increase from baseline in the SOFA scores has the strongest positive correlation with the P-SEP levels, unlike the others (D–F). Spearman’s rank correlation coefficient was used to examine the relationship between the values and SOFA scores. CRP: C-reactive protein, PCT: procalcitonin, P-SEP: presepsin, SOFA score: Sequential Organ Failure Assessment score, Ln: natural logarithm. *SOFA scores were defined based on laboratory data on day 1 in 9 patients and on day 2 in 2 patients (#8 and #11).

We analyzed the relationships of the CRP, PCT, and P-SEP concentrations with the SOFA scores (Fig 1). P-SEP had a significant positive correlation with the SOFA scores (r = 0.89, p < 0.001, Fig 1C). Likewise, PCT significantly correlated with the SOFA score (Fig 1B). When analyzing the relationship of each biomarker with the increase from baseline in the SOFA scores, only P-SEP showed a significant correlation (r = 0.80, p = 0.0025, Fig 1F).

### Changes in the presepsin levels in patients with fungemia

**Fig 2:**
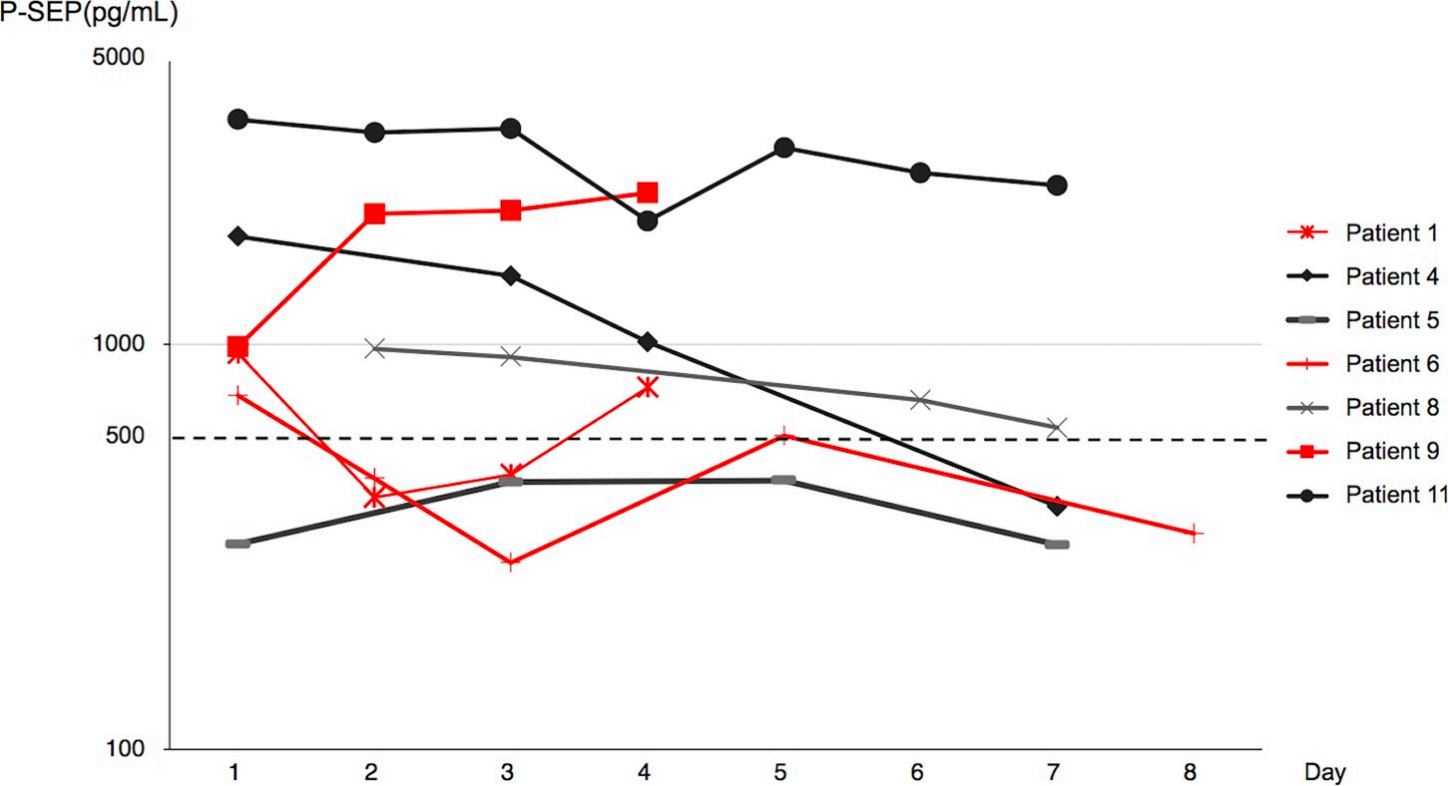
Changes in the presepsin levels in the first week of fungal bloodstream infection. The P-SEP levels increased or increased again after an initial drop in patients who had poor improvement (red lines). In patient #1, the P-SEP levels decreased on day 2 but increased again with bacterial infection on days 3 and 4. In patient #6, P-SEP levels decreased on days 2 and 3, increased again during a catheter-related bloodstream infection on day 5, and returned to normal after removal of the catheter on day 7. Patient #9 died of fungemia on day 4 and had continuous increment in P-SEP levels.

In the 7 patients in whom P-SEP was measured at least 4 times a week after disease onset, the changes in plasma P-SEP levels in the first week are shown in Fig 2. The P-SEP levels decreased promptly with effective treatment but did not decrease or further increase in patients who had poor improvement. In patient #11, who was on hemodialysis, the P-SEP levels remained higher than that in other patients but eventually decreased along with the improvement of fungemia.

### *In vitro* assay

**Fig 3:**
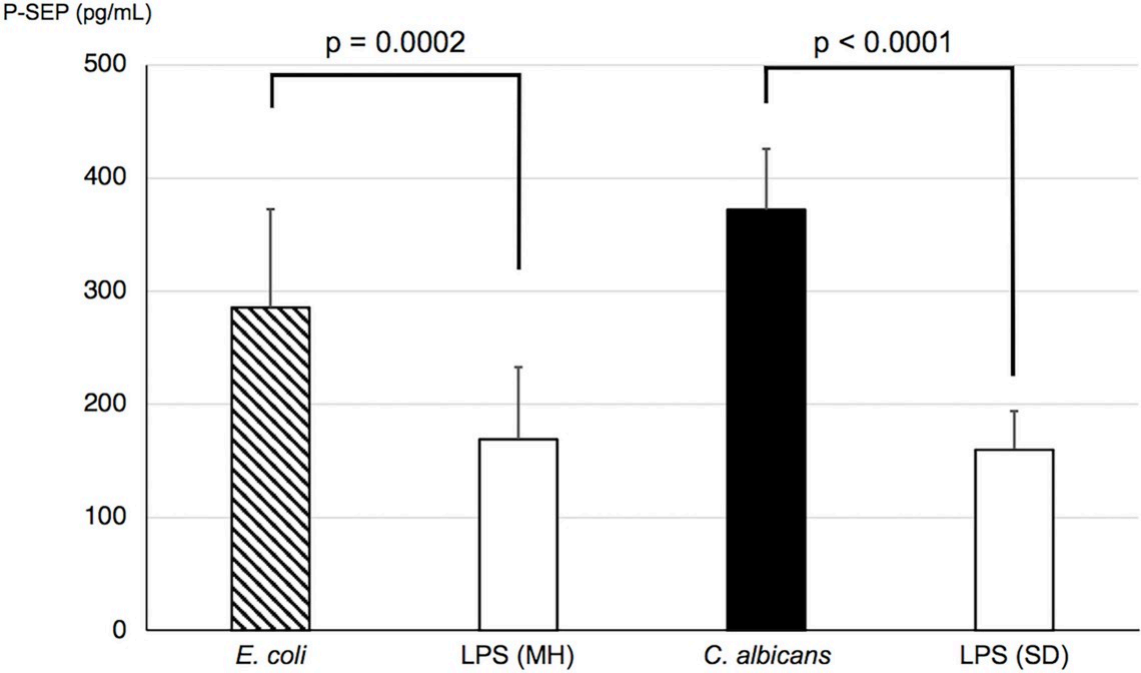
Presepsin levels in the whole blood incubated separately with *C*. *albicans* and *E*. *coli*. Compared with the whole blood sample incubated with LPS, those that were separately cultured with *C*. *albicans* and *E*. *coli* had significant increases in P-SEP levels in the supernatant. The increase in P-SEP levels was not different between the *C*. *albicans* and *E*. *coli* cultures. MH, Mueller–Hinton broth; SD, Sabouraud–Dextrose broth.

In the blood sample incubated with *C*. *albicans*, the P-SEP level in the supernatant significantly increased when compared with that in the negative control (Fig 3). The blood sample incubated with LPS was designated as the negative control or an inflammatory stimulus. Furthermore, the degree of P-SEP elevation was similar between the *E*. *coli* and *C*. *albicans* stimulants. Additionally, *Candida* strains isolated from the patients caused increases in P-SEP (S1 Fig).

## Discussion

P-SEP is widely recognized as a useful biomarker for sepsis. In this study, we found a strong correlation between the P-SEP levels and the SOFA scores in 11 patients with fungal bloodstream infections. In addition, *in vitro* assay by incubation of *C*. *albicans* in human whole blood cells indicated that the viable cells of *C*. *albicans* increased the P-SEP concentrations in a similar degree as that observed in the *E*. *coli* blood culture. Therefore, P-SEP could be a useful biomarker even in fungal infections.

The epidemiology of IFIs has been changing in recent decades due to the development of serologic diagnostic methods and the use of effective antifungal drugs [15, 16]. A multistate point-prevalence survey on health care-associated infections showed that *Candida* spp. was the most common pathogen of bloodstream infections [1], with high rates of morbidity and mortality [2]. Moreover, a delay in the initiation of antifungal treatment increased the mortality risk in patients with candidemia [17, 18]. Therefore, making an appropriate diagnosis as early as possible is important to improve the prognosis of patients with such diseases.

CD14, a precursor of P-SEP, is a 55 kDa glycoprotein that is present on the cell membrane of leukocytes and macrophages. It was identified as a cell surface receptor that binds to the LPS and LPS-binding protein complex and plays an important role in regulating the immune response to endotoxins [19]. Although P-SEP had been reported to be useful for the diagnosis and prognostication of patients with sepsis [6–9], its behavior in fungal infections was unknown.

In the current study, the P-SEP levels were elevated in the clinical samples obtained from 11 patients with fungal bloodstream infection and were associated with the SOFA scores. To date, various biomarkers for fungemia are available in daily clinical practice and include (1,3)-β-D-glucan, mannan, and anti-mannan [20]. Several previous reports have shown that these markers were useful for the diagnosis of candida infections [3, 4] and discontinuation of unnecessary antifungal treatment [21, 22]. However, reports on the value of these serological markers in reflecting the severity and therapeutic response of candida infections are only a few [5]. In addition, PCT did not significantly increase in our patients and had only moderate correlation with SOFA scores. As it has been suggested that PCT does not slightly increase in patients with candidemia [23, 24], P-SEP may be superior as a marker of fungal infections. Our findings showed that P-SEP was associated with the severity of fungemia and could be a useful biomarker in fungal infections, especially those that have invaded the bloodstream.

The biological roles and regulation of the production of P-SEP have not been fully elucidated. Arai et al. reported that monocytes were the main source of P-SEP in humans [12] and showed that P-SEP production was triggered by bacterial phagocytosis or a phagocytic stimulus, rather than an inflammatory stimulus. In this context, we performed *in vitro* experiments and confirmed that the P-SEP levels were more increased in the supernatant of whole blood cells co-cultured separately with *C*. *albicans* and *E*. *coli*, compared with those with LPS stimulation. According to our results, either a bacterial or fungal infection can cause an increase in the P-SEP suggesting that presepsin lacks specificity. Since the increase in P-SEP levels was not different among the pathogens, P-SEP production might be dependent on phagocytosis itself rather than on the kind of pathogen.

Neutropenia and the use of immunosuppressive drugs are risk factors for fungal infections, and, therefore, may also affect the regulation of P-SEP production induced by phagocytosis. Two studies have suggested the usefulness of P-SEP for the diagnosis of febrile neutropenia. Koh et al. reported that at the onset of febrile neutropenia, P-SEP was significantly higher, but PCT was not [25]. Koizumi et al. showed that plasma P-SEP level was strongly associated with the CRP level but not with the absolute WBC count, absolute neutrophil count, and absolute monocyte count [26]. In patient #9 with neutrophil aplasia (neutrophil less than 500/μL), the P-SEP level increased with disease progression. Despite administering a sufficient number of fungi, the *in vitro* assays failed to result in P-SEP increases of more than 500 pg/ml, as observed for severe sepsis. This could be attributed to the differences in the absolute count of monocytes, macrophages, and granulocytes and the absence of tissue monocytes and macrophages. Because presepsin or sCD14 subtype is believed to derive from CD14 on the surface of monocytes, macrophages, and granulocytes [12], these results may suggest that P-SEP elevation in plasma is derived not only from circulating monocytes but also from tissue monocytes and macrophages. During the course of patient #7, the effect of corticosteroids was lower on P-SEP than on the CRP. Therefore, immunosuppressive drugs may have little effect on the regulation of P-SEP expression.

There are some factors that may affect the P-SEP concentrations in patients without infection. For example, patients with renal failure, especially those on hemodialysis, were reported to have relatively high P-SEP levels even in the absence of infection [27, 28]. Unfortunately, these patients have a high risk of infections, including those of invasive candidiasis [29]. In one hemodialysis patient in this study, P-SEP remained high. Therefore, in patients with chronic kidney disease or those on hemodialysis, it would be necessary to set different cutoff values for P-SEP; moreover, the value of P-SEP should be interpreted carefully when such patients acquire severe infections, including fungemia.

There were some limitations in the present study. First, it was a single-center study; the possibility of unintentional selection and hospital biases could not be fully excluded. Second, the number of cases was insufficient to verify the usefulness of P-SEP as a biomarker. Moreover, presepsin levels were not serially followed for all the included patients. The correlation between presepsin levels and the outcomes was not always much significant. Given the very limited number of included patients, we could not extrapolate if this was due to a presepsin performance issue or any other study confounding factors.

In conclusion, our findings suggested that plasma P-SEP can increase in both fungemia and bacterial infections and that the levels of P-SEP can reflect the severity of fungal infections. Our results need to be confirmed with studies involving larger cohorts, hopefully in a prospective manner from multiple institutions, to state that *Candida* species-invasive infections are associated with the release of presepsin in blood, probably originating from macrophages and monocytes. *In vitro* assay confirmed that the viable cells of *C*. *albicans* could increase the P-SEP to levels that are similar with that seen with *E*. *coli*. It would be interesting and valuable to conduct experiments with blood from patients suffering from different diseases and receiving different immunosuppressive drugs to establish P-SEP as a useful marker for sepsis due to fungal infection.

## Supporting information

S1 Table Initial laboratory findings and use of immunosuppressive drugs in 11 patients with fungal bloodstream infection. (DOCX)

S1 Fig Presepsin levels in the whole blood incubated with the *Candida* species. (DOCX)
