# Supplementary material for: Increased presepsin levels are associated with the severity of fungal bloodstream infections
Source: PLoS One. 2018 Oct 31;13(10):e0206089. doi: 10.1371/journal.pone.0206089 (PMC6209217; doi:10.1371/journal.pone.0206089)
Supplement: S1 Table — (DOCX) [file pone.0206089.s001.docx]

**Table S1. Initial laboratory findings and use of immunosuppressive drugs in 11 patients with fungal bloodstream infection**

| case | Sepsis or not^*^ | Presepsin  (pg/mL) | Procalcitonin  (ng/mL) | Neutrophil  count (/µL) | Lymphocyte  count (/µL) | Monocyte  count (/µL) | Immunosuppressive drugs prescribed |
| --- | --- | --- | --- | --- | --- | --- | --- |
| 1 | Sepsis | 950 | 0.16 | 5202 | 467 | 399 | N/A |
| 2 | Sepsis | 12074 | 11.5 | 16556 | 626 | 104 | N/A |
| 3 | Non-sepsis | 443 | 0.05 | 4260 | 798 | 399 | Prednisolone 2 mg/day |
| 4 | Sepsis | 1848 | 7.08 | 12727 | 548 | 411 | N/A |
| 5 | Non-sepsis | 321 | 0.16 | 2349 | 620 | 131 | Prednisolone 5 mg/day |
| 6 | Non-sepsis | 748 | 0.57 | 4870 | 1429 | 412 | N/A |
| 7 | Sepsis | 9367 | 0.47 | 14303 | 6026 | 2183 | Prednisolone 12 mg/day |
| 8 | Non-sepsis | 975 | 0.4 | 6125 | 638 | 312 | N/A |
| 9 | Sepsis | 988 | 31.72 | 19 | 820 | 10 | Tacrolimus 2 mg/day |
| 10 | Non-sepsis | 793 | 0.12 | 7450 | 360 | 208 | Prednisolone 20 mg/day |
| 11 | Sepsis | 3591 | 16.52 | 23825 | 1797 | 208 | N/A |

Laboratory data on day 1 in 9 patients and on day 2 in 2 patients (patients #8 and #11) are shown.

^*^ Sepsis was defined with an increase of 2 or more points from the baseline in SOFA score..

N/A, not applicable.
