# Supplementary material for: Increased presepsin levels are associated with the severity of fungal bloodstream infections
Source: PLoS One. 2018 Oct 31;13(10):e0206089. doi: 10.1371/journal.pone.0206089 (PMC6209217; doi:10.1371/journal.pone.0206089)
Supplement: S1 Fig — (DOCX) [file pone.0206089.s002.docx]

**Figure S1. Presepsin levels in the whole blood incubated with the *Candida* species**


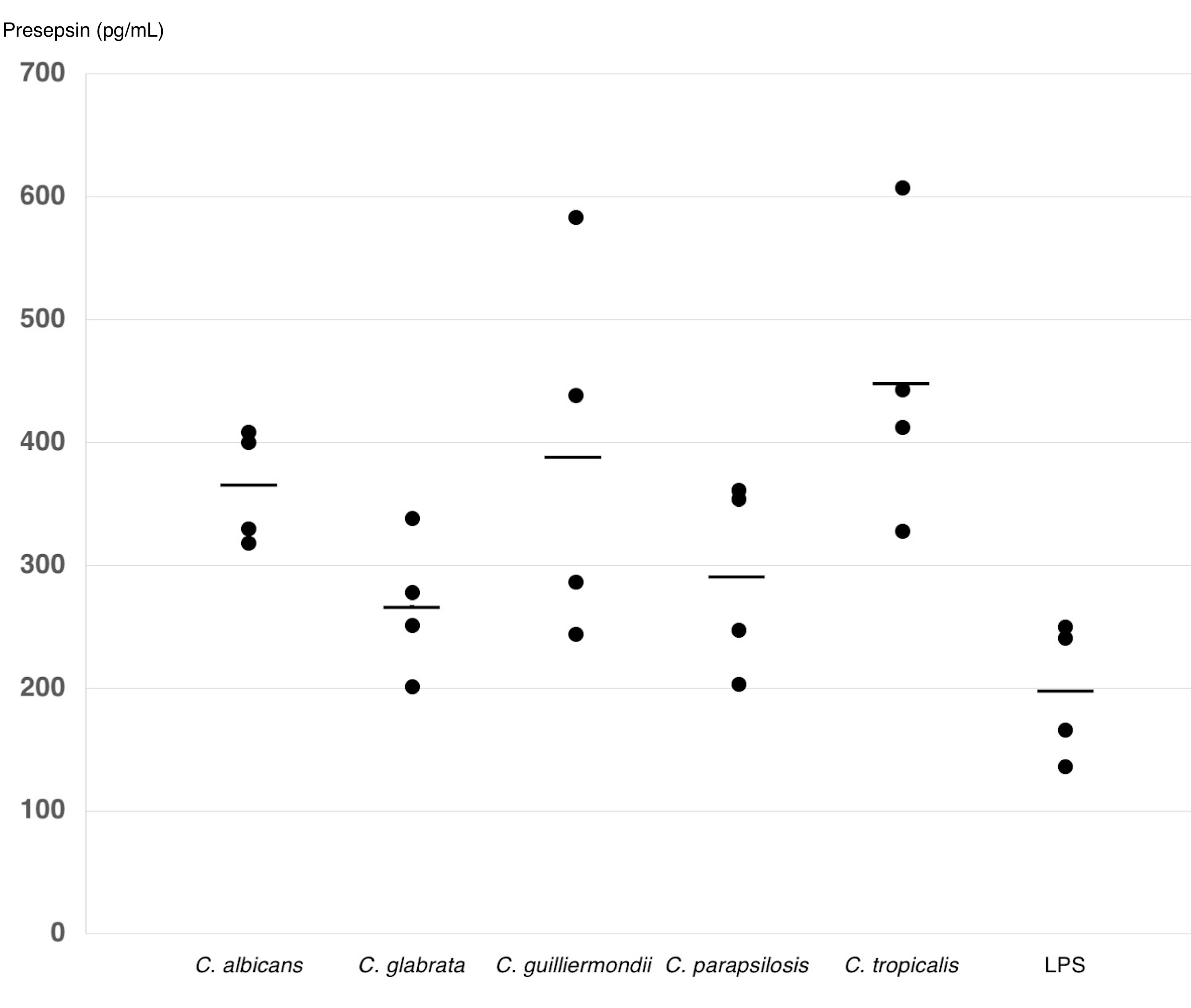


Compared to LPS, all the *Candida* species tended to increase presepsin levels after coculture.

The white blood cell count (WBC) and fraction of the volunteers were as follows: WBC, 5770 ± 1893 /µL; Neutrophil, 3212 ± 860 /µL; Lymphocyte, 2048 ± 884 /µL; Monocyte, 314 ± 126 /µL; Eosinophil, 158 ± 132 /µL; and Basophil, 38 ± 20 /µL.
